# Viable *Neisseria meningitidis* is commonly present in saliva in healthy young adults: Non-invasive sampling and enhanced sensitivity of detection in a follow-up carriage study in Portuguese students

**DOI:** 10.1371/journal.pone.0209905

**Published:** 2019-02-11

**Authors:** Fernanda Rodrigues, Hannah Christensen, Begonia Morales-Aza, Paulina Sikora, Elizabeth Oliver, Jennifer Oliver, Jay Lucidarme, Robin Marlow, Luís Januário, Adam Finn

**Affiliations:** 1 Hospital Pediátrico, Centro Hospitalar e Universitário de Coimbra, Coimbra, Portugal; 2 Faculdade de Medicina, Universidade de Coimbra, Coimbra, Portugal; 3 School of Population Health Sciences, University of Bristol, Bristol, United Kingdom; 4 School of Cellular and Molecular Medicine, University of Bristol, Bristol, United Kingdom; 5 Meningococcal Reference Unit, Public Health England, Manchester, United Kingdom; RIVM, NETHERLANDS

## Abstract

**Introduction and aims:**

Improved sensitivity and efficiency of detection and quantification of carriage of *Neisseria meningitidis* (Nm) in young people is important for evaluation of the impact of vaccines upon transmission and associated population-wide effects. Saliva collection is quick, non-invasive and facilitates frequent sampling, but has been reported to yield low sensitivity by culture. We re-evaluated this approach in a follow-up cross sectional study using direct and culture-amplified PCR.

**Material/Methods:**

In April 2016 we collected paired oropharyngeal swabs (OPS) and saliva samples from 1005 healthy students in Portugal into STGG broth and stored them at -80°C until DNA extraction and batched qPCR analysis. Samples were also cultured on GC agar plates for 72h and PCR done on DNA extracts from overall growth. Nm isolates were also sought from a selection of 50 samples. qPCR amplification targets were superoxide dismutase *sodC* and capsular locus/genogroup-specific genes (B, C, W, X and Y) and, for cultured isolates only, *porA*. Cycle threshold values of ≤36 were considered positive.

**Results:**

556 tests (460 samples, 363 subjects, 36.1%) were positive for Nm (*sodC*) and 65 (45, 36, 3.6%) for MenB. More salivas were positive by direct *sodC* qPCR (211, 21.0%) than OPS (126, 12.5%) but fewer were positive by culture-amplified qPCR (94 vs. 125). For both sample types, many that were negative on direct qPCR came positive on culture-amplification and Nm was consistently isolated from salivas in which culture amplified the PCR signal. Using both methods on both samples yielded 36.1% Nm and 5.5% encapsulated Nm carriage rates while direct qPCR on OPS alone detected 12.5% and 2.2%.

**Conclusions:**

Detectable MenB carriage rates (2.9%) were lower than 4 years earlier (6.8%) in this population (p = 0.0003). Viable meningococci were often present in saliva. Although evidence of encapsulated Nm was less frequent in saliva than OPS, collection is more acceptable to subjects allowing more frequent sampling. Use of culture-amplification increases detection sensitivity in both sample types, especially when combined with direct PCR. Combining these samples and/or methodologies could greatly enhance the power of carriage studies to detect the impact of vaccines upon carriage and transmission.

## Introduction

Carriage of *N*. *meningitidis* (Nm, meningococcus) has been the subject of increased recent study due to recognition that, although licensed on the basis of immunogenicity and inferred direct protection in recipients, the principal and most easily-exploited mechanism of action of meningococcal conjugate vaccines is their impact on transmission at the population level [1] and consequent interest in whether novel protein antigen vaccines may have analogous effects. We have reported advances in sampling and laboratory methodology which simplify the logistics and greatly reduce the costs of performing carriage studies and have used them to describe a previously unknown distribution of colonisation density, heavily skewed with a large majority of carriers having small numbers of bacteria and a minority of around 10–15% carrying 2–4 orders of magnitude more organisms in a cross-sectional population sample [2].

Detection of nasopharyngeal bacteria in saliva samples has been reported [3], but the only previous published study of this sampling approach to studying meningococcal carriage concluded that it was not useful [4]. Nevertheless, mouth to mouth contact is an established risk factor for meningococcal carriage and suggests that viable organisms may be present in the saliva of carriers [5]. Obtaining oropharyngeal and nasal swabs, the standard methods for studying carriage of Nm and other bacterial residents of the upper respiratory tract, are moderately invasive techniques. Many people do not like the experience of having them taken and consequently studies involving numerous and/or frequent swabs are difficult to perform as subjects quickly lose enthusiasm for participation. In contrast, obtaining samples of saliva is quick, easy and non-invasive. Subjects can readily provide the samples without assistance. This approach, if accurate, therefore has the potential to permit much more intensive studies of the biology of colonisation which could help overcome issues with sampling error and have greater power to detect changes in rates of acquisition and in duration and density of carriage.

We hypothesised that molecular detection using quantitative polymerase chain reaction (qPCR) might have the potential to detect Nm in saliva samples more readily than the attempts to use culture, reported previously [4]. Introduction of a culture step prior to DNA extraction and PCR might both indicate the presence of viable organisms when signals were compared to those from PCR of uncultured samples and might increase the sensitivity of detection among the large number of low density carriers if viable bacteria were present, as reported recently for an overnight incubation step following sampling [6]. We conducted a study in healthy students at the University of Coimbra in Portugal, a population we have studied previously [2, 7]. By taking paired oropharyngeal swabs (OPS) and saliva samples from each subject, we were able to compare the sensitivity of both techniques and also evaluate to what extent it could be enhanced by analysing both samples taken at the same time. The results, presented here, establish clearly that saliva sampling has the potential to enhance the ease and sensitivity of detection of Nm greatly. We therefore propose that it should become at the least an adjunct to and, when frequent samples are needed, an alternative to OPS in future carriage studies.

## Materials and methods

### Subjects

Undergraduate students in all academic years at the University of Coimbra, Portugal, were invited to participate via the students’ website, and were recruited after lectures. Demographic and clinical data were collected for all subjects, as summarised in the results. The study was approved by the Ethics Committee of Coimbra School of Medicine (CE-6/2012) and all subjects provided written informed consent.

### Sampling methods

Between 18–28 April 2016, OPS and saliva samples were collected by trained nurses, following standard operating procedures:

- OPS: sterile double-headed swabs (MW821DC Medical Wire & Equipment, Wiltshire, UK) were inserted through the open mouth, touching the tips against one pharyngeal tonsillar fossa and then passed in an upwards semi-circular motion over the soft palate to the opposite side.
- Saliva: sterile foam polygon swabs (RML120-905 Rocialle, South Wales, UK) were put into participants’ mouths where they stayed until saturated with saliva. The foam tip was then removed from the stick and placed into the barrel of a sterile syringe and the saliva squeezed out directly into a cryovial using the syringe plunger.

OPS and saliva samples were placed in 1.5mL and 1mL skim milk-tryptone-glucose-glycerol (STGG) broth, respectively and stored at -80°C until analysis. Viability through this freeze thaw step is well preserved as previously described [2]. The second head of each OPS was placed into RNA preservation medium for other studies.

### Laboratory methods

#### Culture and identification

Strains and isolates used in the specificity panel were cultured using standard techniques as previously described [8]. For detection of Nm from swab and saliva samples, 100μL broth was inoculated onto GC agar plates (E&O Laboratories, Cumbernauld, UK) and after incubation at 37°C in 5% CO_2_ for 72h, all growth from each plate was removed using a plastic loop into 1mL of Trypticase Soy Broth (TSB) (Becton Dickinson GmbH) with 15% glycerol and stored frozen at -70°C. A selection of these samples were later thawed and streaked onto CBA agar plates (PB0122A Columbia agar with horse blood, Oxoid) and cultured at 37°C in 5% CO_2_ overnight (16–18 h). Colonies suspected to be Nm were then sub-cultured onto further CBA plates and pure isolates again stored in 1 mL TSB with 15% glycerol at -70°C. Isolates were Gram stained and tested for oxidase activity using standard methods.

#### DNA extraction, PCR and sequencing

Automated extraction of nucleic acids from swab and saliva samples (STGG broth), products of culture and pure isolates was performed followed by qPCR analysis for the superoxide dismutase gene *sodC* [9] *porA* (isolates only) [10] and capsular locus/genogroup-specific loci as previously reported [2] with the following modifications: cycle threshold (C*t*) values ≤36 were considered positive in the assays (≤35 for *porA* assay), three dilutions of DNA extracts of each target reference strain were used as positive controls and DNA extracts of STGG broth and L6 lysis buffer as negative reagent controls. C*t* values (*sodC*) were converted into bacterial gene density values (in gene copies/mL) as previously described [2].

The specificity of the *sodC* PCR assay for the detection of Nm [9] was reconfirmed by running DNA extracts from a range of reference strains and upper respiratory tract isolates as shown in S1 Table.

Whole genome sequencing was performed as previously described [11]. Briefly, DNA was extracted from cultures using the Wizard Genomic DNA Purification Kit (Promega Corporation). One hundred base pair paired-end sequence analysis was performed on the Illumina HiSeq 2000 and contigs were assembled *de novo* using Velvet assembler (v1.2.08) [12]. Assembled contigs were submitted to the PubMLST Neisseria database (https://pubmlst.org/neisseria/).

### Statistical analysis

The number of positive swabs obtained using different detection methods was calculated for all samples and for particular capsular groups. For the descriptive demographic analysis, the age of students was calculated from their date of birth using the mid-point of the sampling period. Where students had recorded taking an antibiotic, but the product name recorded was either not an antibiotic or did not exist, these students were assumed not to have taken an antibiotic. Comparisons of proportions were made using the Chi-squared test, of independent density using the Mann-Whitney–U and paired densities with the Wilcoxon Signed Rank Test. Analyses were performed using Stata v14.1 and R 3.5.1.

## Results

1,006 students consented in the study; the mean age of these students was 21 years (range 16 to 49), 9.3% were smokers, with 27.2% being exposed to smoking at home. Full demographic characteristics of the students are shown in Table 1 and are provided to facilitate comparison with previously published reports from our group and others. Based on coverage figures at the time, the large majority of these students are likely to have received MenC conjugate vaccine in the catch-up programme ten years earlier. One student was excluded from the analysis because sampling was incomplete leaving 1,005 subjects with results for both OPS and saliva by both direct and culture amplified qPCR.

**Table 1:** Demographic characteristics of the sampled students with complete laboratory data (n = 1005).

Category		N
Age group	15–19	289
	20–24	594
	25–29	66
	30–34	12
	35–39	2
	40–44	2
	45–49	1
	Data missing	39
Taken antibiotics in the previous month	No	897
	Yes	107
	Data missing	1
Currently taking antibiotics	No	980
	Yes	21
	Data missing	4
Smoker	No	911
	Yes	93
	Data missing	1
Exposed to smoking at home	No	724
	Yes	271
	Data missing	10
Lives in a student residence	No	849
	Yes	150
	Data missing	6
Number of people individual is living with in household	0	31
	1–4	842
	5–9	92
	10+	33
	Data missing	7

The OPS direct *sodC* qPCR results showed an overall carriage rate of 12.5% with a distribution of detected capsular groups that was predominantly B with smaller numbers of the other groups that were tested for (Table 2).

**Table 2:** Numbers and percentages of oropharyngeal swab (OPS) and saliva samples positive (C*t* values ≤36) by direct and culture-amplified qPCRs for *N*. *meningitidis* (*sodC*) and each of 5 capsular groups.

	All Nm (%)	B (%)	C (%)	W (%)	X (%)	Y (%)	BWXY (%)
OPS direct	126 (12.5)	17 (1.7)	0	1 (0.1)	2 (0.2)	2 (0.2)	22 (2.2)
OPS culture-amplified	125 (12.4)	29 (2.9)	0	3 (0.3)	2 (0.2)	5 (0.5)	39 (3.9)
OPS direct & culture-amplified combined	197 (19.6)	31 (3.1)	0	3 (0.3)	4 (0.4)	6 (0.6)	44 (4.4)
Saliva direct	211 (21.0)	7 (0.7)	0	0	5 (0.5)	1 (0.1)	12* (1.2)
Saliva culture-amplified	94 (9.4)	12 (1.2)	0	1 (0.1)	3 (0.3)	1 (0.1)	17 (1.7)
Saliva direct & culture-amplified combined	263 (26.2)	14 (1.4)	0	1 (0.1)	6 (0.6)	2 (0.2)	22* (2.2)
Both samples and both techniques combined	363 (36.1)	36 (3.6)	0	3 (0.3)	9 (0.9)	8 (0.8)	55* (5.5)

*one saliva sample was positive for both X and Y by direct qPCR.

Samples from the three subjects who were positive for capsular group W were cultured and isolates were subjected to whole genome sequencing. All were ST-11 complex isolates belonging to lineage 11.1. Two of these clustered with South African invasive isolates from 2003–2013 while one belonged to the South American strain sublineage and was closely related to the ‘original UK strain’ that emerged in the UK in 2009 [7, 13, 14]. Only one subject was found to be carrying more than one capsular group, namely X and Y (both detected from a saliva sample by direct PCR). Overall detection rates of Nm and of the selected capsular groups in each of the sample types and by both direct and culture-amplified qPCR are also shown in Table 2 along with rates when the results of the two techniques are combined for each sample type and when both techniques and both samples are all combined for each subject. The overall detection rate was 36.1%, nearly 3 times higher than that by direct analysis of OPS alone. The use of two sample types and both direct and culture-amplified qPCR techniques also enhanced the overall detection rates of encapsulated Nm groups B, Y, X and W but not group C, of which none were detected in this study.

**Fig 1:**
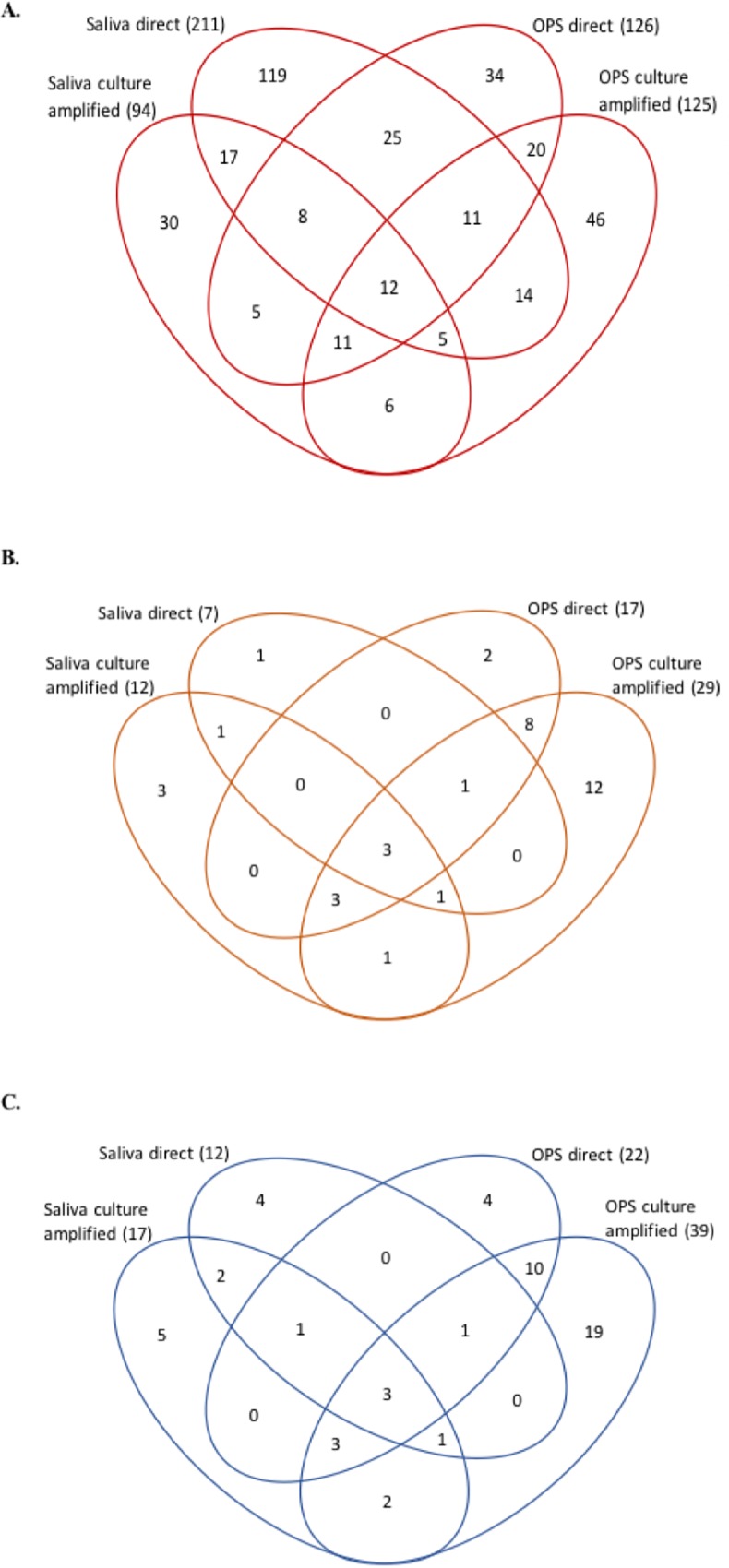
PCR positivity for all meningococci (*sodC*) (A), capsular group B meningococci (B) and capsular groups B, W, X and Y meningococci combined (C) determined by different methods. Numbers of positives among 1005 subjects (n = 363, 36 and 55, respectively) are shown in each area of the Venn diagrams according to their distribution among each sample and PCR technique used. There were no cases of MenC and 1 subject carried both X and Y. OPS–oropharyngeal swab.

The distribution of samples positive by the generic Nm *sodC* qPCR assay among the two sample types and two techniques is shown in full in Fig 1A. In the majority of the 263 subjects in whom Nm was detected in saliva, this positive result was by direct PCR only (169, 64%), with only 94 (36%) positive by culture-amplified PCR of which 42 (16%) were positive by both techniques. This contrasts with the 197 positive OPS samples in which the respective figures were 72 (37%) direct only, 125 (63%) culture-amplified PCR of which 54 (27%) were positive by both techniques. As can be inferred from the overall higher rates of detection using both samples, the number of subjects in whom Nm was detected in only one sample (saliva 166, OPS 100) was greater than the number in whom the bacteria were detected in both (97) and in only 12 individuals was Nm detected in both samples by both PCR techniques. The equivalent data for capsular group B Nm and for all 4 detected capsular groups combined are shown in Fig 1B and 1C respectively. The single sample and technique combination which detected the most potentially pathogenic strains was culture-amplified qPCR on OPS samples. However, a considerable number of additional capsule-positive samples were found both on direct qPCR of OPS samples and in saliva by both techniques, even though the increases in sensitivity achieved by adding saliva were smaller for the encapsulated strains than for Nm overall.

**Fig 2:**
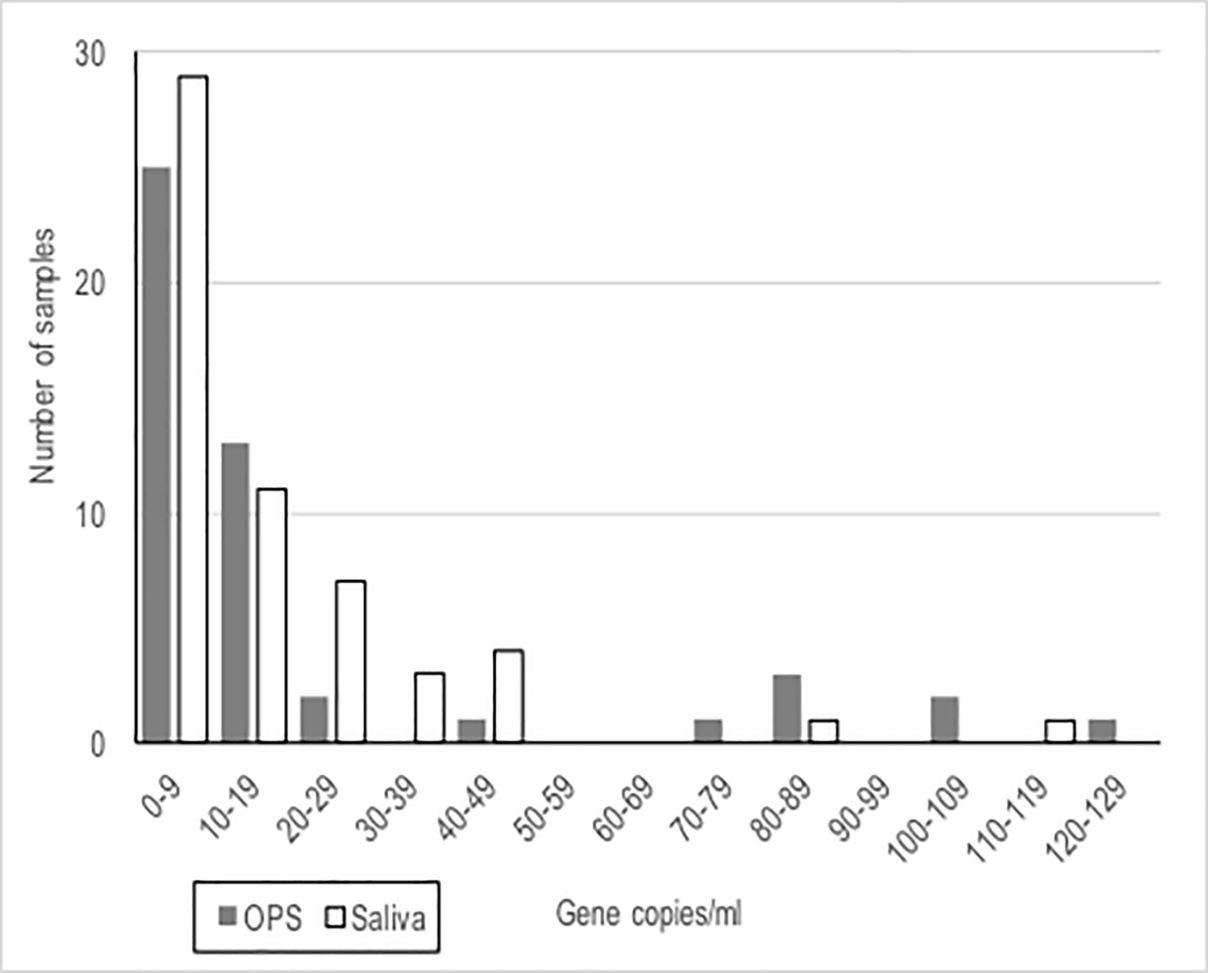
Density of *N*. *meningitidis* for the 56 samples positive by direct *sodC* PCR in both saliva and OPS samples (the OPS values for the 5 samples with >140 gene copies/mL, densities of which can be seen in Fig 3, are omitted from the figure to permit the density distributions to be more clearly seen). OPS–oropharyngeal swab.

**Fig 3:**
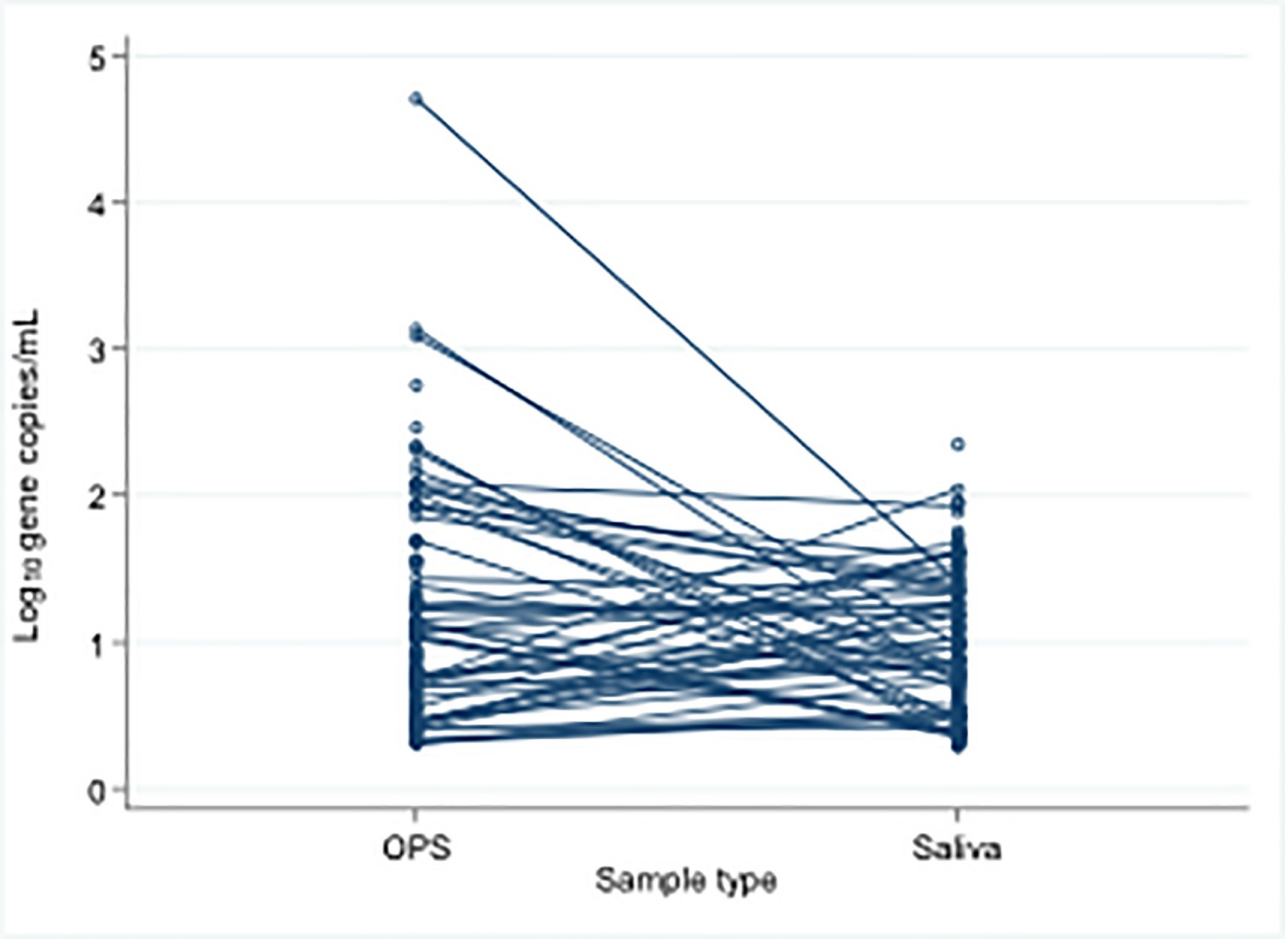
Density of *N*. *meningitidis* in all the saliva (n = 211) and OPS (n = 126) samples positive by direct *sodC* qPCR (connected lines denote positive sample pairs from the same individuals). OPS–oropharyngeal swab.

The median bacterial density when positive by direct qPCR was slightly but significantly higher in OPS samples (n = 126) than in saliva (n = 211) (p = 0.008) with a density distribution skewed towards low density in both sample types (Fig 2) as we reported previously for OPS [2]. Among the 56 subjects that were direct *sodC* qPCR positive in both OPS and saliva samples, median values were similar (p = 0.11), although, in many of these individuals, density was higher in saliva than in OPS (Fig 3).

**Fig 4:**
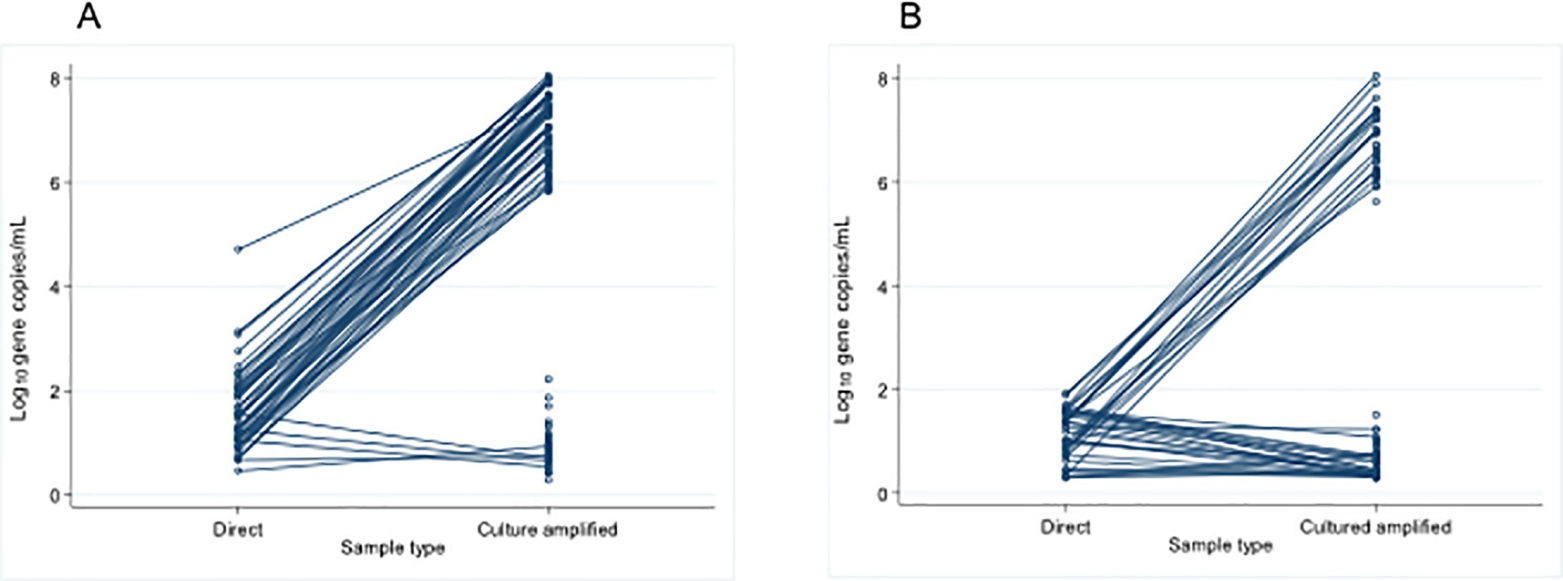
Density of *Neisseria meningitidis* in OPS (A) and saliva (B) samples by direct qPCR (n = 126, 211) and cultured amplified (n = 125, 94) qPCR. Connected lines show samples positive by both methods; also shown as unconnected points are samples negative by direct qPCR but positive by culture-amplified qPCR.

The densities of Nm gene copies in both OPS and saliva culture-amplified samples fell into two completely distinct populations (Fig 4). Those with very high (range 4.15x10^5^ to 1.16x10^8^ gene copies/mL) densities (73 OPS, 42 saliva) are, we surmise, the products of active proliferation of viable bacteria in culture and, in subjects whose direct qPCR assay in the same sample was also positive, the fold-rise in gene copy number ranged from 6.11x10^2^ to 4.22x10^6^, confirming this. The samples with lower densities (range <60 to 169.11 gene copies/mL), a range similar to those seen in the direct qPCR assay, may represent bacterial DNA in the absence of viable bacteria. For such samples, when both direct and culture-amplified assays were positive, the densities were similar or slightly lower in the latter assay, which involves a times 10 dilution.

In order to confirm that saliva samples inferred to contain viable Nm on the basis of culture-amplification of the *sodC* PCR signal, really contain these bacteria, as shown previously for throat swab samples [2], the products of culture on selective agar plates from the 42 saliva samples which were positive by *sodC* qPCR at high density and 8 of those which were positive at low density (Fig 4B) were re-cultured on non-selective plates and all colonies observed which had the characteristic appearance of Nm were then sub-cultured and identified by Gram stain, oxidase testing and qPCR of DNA extracts for both *sodC* and *porA* genes. Nm was successfully cultured and positively identified by all these criteria from all 42 high density samples but from none of the low density samples.

**Fig 5:**
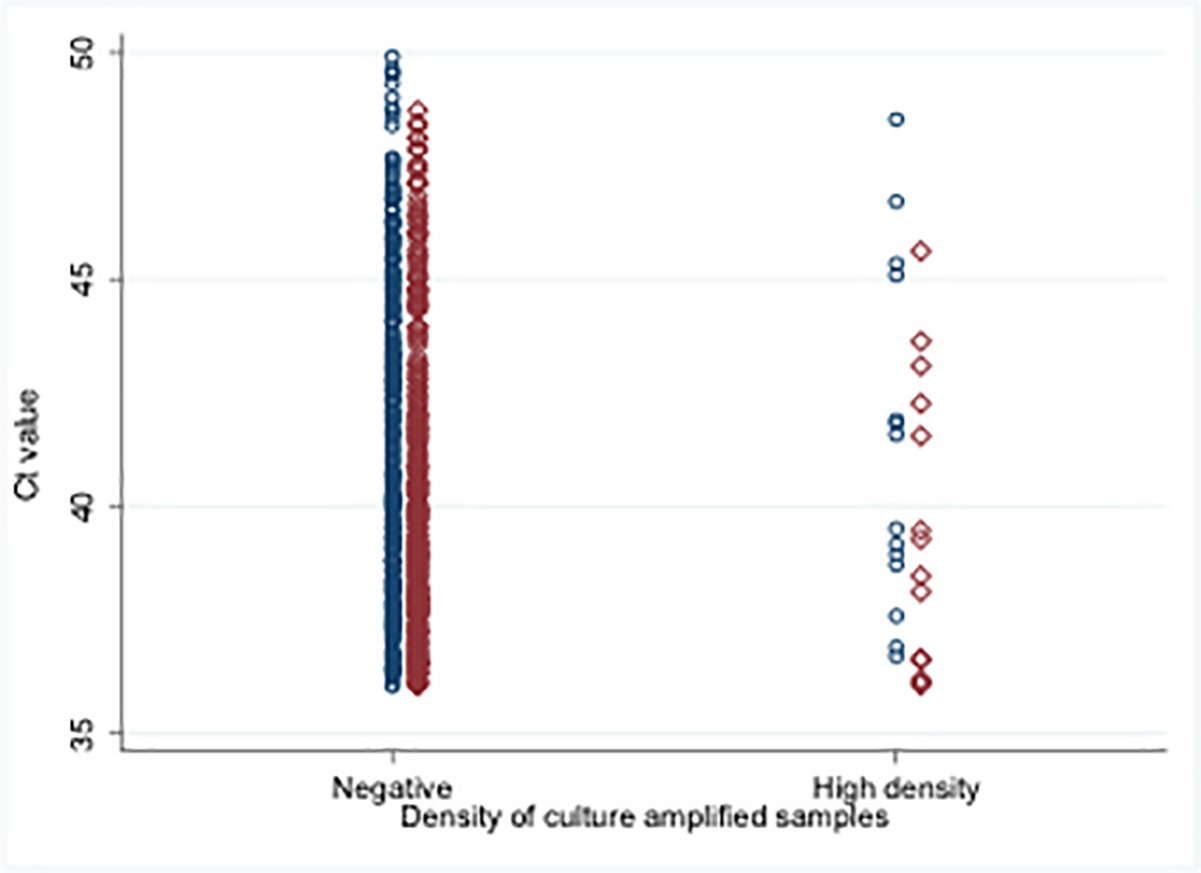
A subset of samples (OPS (o) and saliva (◊)) with direct qPCR signals in the C*t* range 36–50 (thus designed negative) are shown. On the left are those which showed no culture-amplified qPCR signal at all up to 50 cycles and on the right those which showed high density (>10^5^ gene copies/mL) by the culture-amplified qPCR assay (see Fig 4) indicating the presence of viable Nm. Both groups demonstrate a similar wide range of C*t* values.

Some of the samples with high density (as defined above) culture-amplified qPCR results, which therefore contain viable Nm, did not produce positive direct qPCR results (i.e. had C*t* values >36). Knowing that the latter results were false negatives, we examined their C*t* values alongside those of other samples also negative by direct qPCR but which had no signal at all on culture-amplified qPCR and were likely, therefore, to contain no viable Nm (Fig 5). The ranges and distributions of late C*t* values observed for these two distinct sets of samples were similar.

## Discussion

This follow up cross sectional study conducted 4 years after the first meningococcal carriage study done in Portugal [2, 7], in the same student population, shows a similar rate of overall Nm carriage when the results using identical methodology are compared (direct *sodC* qPCR on throat swabs collected into STGG broth), with 12.5% in 2016 and 14.5% in 2012 [2] (p = 0.3). In the earlier study, capsular groups C, W, Y, and X Nm were detected in small numbers while the former remained undetected in the more recent study, despite the passage of 4 more years, and potential consequent reductions in population immunity, since the large meningococcus group C conjugate vaccine catch up programme performed in the country in school aged children in 2006. No increase in W carriage was seen despite contemporary reports of increasing rates of invasive disease due to this capsular group in several European countries [15–17]. However, on this occasion, one of the three group W isolates was genetically similar to a distinct strain of the invasive South American strain sublineage that was first observed to be circulating in the UK around 2009 [13]. In both studies, the mostly commonly detected capsular group in carriage was B which is also the main cause of invasive disease in the country [18]. The observed carriage rates of group B were 2.7% directly from the OPS sample and 6.8% following culture-amplified qPCR in 2012 and, using the same two approaches in 2016 were 1.7% (p = 0.24) and 2.9% (p = 0.0003), respectively (Table 2). There are no obvious differences between the demographic characteristics of the two groups of subjects to explain this lower rate of detection. The second study took place about one calendar month earlier in the year than the first, just before an annual week of celebrations and festivities which preceded the earlier study. Although close social contact is a known risk factor for meningococcal carriage [5], this cannot easily explain a selective drop in carriage rates of group B with limited evidence of such a trend for other capsular groups or overall Nm carriage rates. Accordingly, we may have observed a genuine drop in carriage rates of group B between 2012 and 2016 in this population, a possible change which ought to be studied further alongside surveillance of cases of invasive disease.

The known association between meningococcal carriage and mouth to mouth kissing [5] suggests that the organism may be transmitted in saliva, although a previous attempt to demonstrate this was unsuccessful [4]. In the majority of the 211 study subjects reported here, in whom *sodC* was detected in saliva, there was no evidence of viable bacteria, since culture-amplified PCR was negative, but 42 had high-density positive results by the latter technique, providing strong evidence of live salivary Nm in 4.2% of those studied which was subsequently reconfirmed by culture. Overall, more subjects in this study had evidence of *sodC* in saliva than in OPS. From this, it can be concluded that Nm transmission via saliva can occur. Presence of Nm DNA either in OPS or saliva, even in the absence of viable bacteria, can be interpreted as evidence of current or recent colonisation and is therefore potentially valuable information in a carriage study. Nevertheless, it is clear that Nm bearing the capsular loci tested for in this study are less likely to be detected in saliva than in OPS (Fig 1C).

Since identification of colonies from culture plates by Gram stain and oxidase testing was not undertaken in this study, (apart from the 3 samples positive for group W that were then sequenced and the 42 saliva samples with high density *sodC* PCR), the results do not permit comparison of performance of that approach to identification of Nm, used in most previous studies of Nm carriage and still a commonly-used method, with molecular detection as used here. However, in a previous study in this population [2] we showed generally similar and higher overall and capsular positive detection rates by direct and culture-amplified PCR, respectively, as compared to traditional culture methods. In another recent study in 16–18 year old healthy school students in Bristol, among 1813 oropharyngeal swabs analysed by traditional culture, direct and culture-amplified *sodC* PCR, there were 320 positive by at least one of the 3 methods of which only one was positive only by culture, the other 133 culture-positives also being positive by culture-amplified PCR (unpublished data). Accordingly, while an important benefit of obtaining, speciating and saving pure isolates when performing cultures from carriage studies relates to additional characterisation that can then be performed, including whole genome sequencing, such expensive and time-consuming processing can reasonably be reserved for the minority of samples that test positive by one or other of the PCR methods as long as swabs and/or saliva samples have been collected into glycerol containing storage media such as STGG so that pure isolates can be prepared when needed [2].

The distribution of meningococcal DNA densities among carriers appear similar in OPS and saliva samples with most being low. However, the range of densities recorded in OPS samples extended higher than seen in saliva (Fig 3). Although on average, bacterial gene copy numbers are slightly higher in the throat, many individuals in whom direct qPCR was positive in both samples had higher values in their saliva than in their OPS. Taken together with the many subjects in whom evidence of Nm was only found in saliva (Fig 1A), this demonstrates the possibility of sampling error and false negatives and emphasises the value of taking this sample, in addition to throat swabs, to maximise the sensitivity of any carriage study.

The two very distinct populations of high- and low-density culture-amplified qPCR results for both sample types (Fig 4) seems to represent those with and without viable Nm present, respectively. However, the presence of high density *sodC* DNA following culture amplification did not reliably predict a low C*t* value among samples that generated amplification curves in the range 36–50 cycles. Accordingly, we still do not have a simple and reliable way to determine which of these results of direct qPCR analysis, currently designated “negative” may actually be true positives.

A possible limitation to the results presented here arises from the imperfect sensitivity and specificity of amplifiable *sodC* for identification of Nm. Although *sodC* PCR has been shown to be highly specific and sensitive for Nm isolates [9], it has been suggested that this gene may originally have been acquired by Nm by horizontal transfer from strains of haemophilus species [19] and that the gene could therefore potentially be detected from such strains [20] and may also not be present in all Nm strains [21]. However, respiratory carriage of *Haemophilus influenzae* is unusual in this age group [22] and we could not find the gene in multiple strains of the species obtained from the same population (S1 Table). All the results of the capsular group PCRs are expected to be specific. Only two samples that were positive by genogrouping PCR were negative by direct *sodC* PCR, one OPS sample and one saliva, from two different subjects both positive for MenX. False positive or negative results using the *sodC* assay in this study are therefore unlikely to have had significant influence on the overall findings.

Overall, the results of this study show that viable meningococci can be present in saliva of carriers, where evidence of the organism can also be detected using direct qPCR more frequently than in OPS. Although saliva testing has lower sensitivity for detecting genogroupable strains, the sampling methodology is much less invasive and allows much more frequent and numerous samples to be taken than would be acceptable to most subjects for OPS. Taking saliva samples in addition to OPS in meningococcal carriage studies should significantly increase the sensitivity of detection with modest additional effort and cost. Culture-amplified PCR increases sensitivity of detection in both samples when combined with direct PCR detection. Use of these methodologies should greatly enhance the power of carriage studies to detect the impact of vaccines upon carriage and transmission.

## Supporting information

S1 Table Specificity panel for *sodC* PCR. DNA extracts (4 serial 10x dilutions from each) from cultures of the strains listed demonstrated no signal up to 50 cycles in all cases. Conversely, similar dilutions of extracts from *N*. *meningitidis* strains ATCC 53417(A), BAA-335(B), 53414(C), 53419(D), 35559(W), 35560(X) and 35561(Y) consistently amplified after 25 cycles or less.
(DOCX)
