# Supplementary material for: Viable Neisseria meningitidis is commonly present in saliva in healthy young adults: Non-invasive sampling and enhanced sensitivity of detection in a follow-up carriage study in Portuguese students
Source: PLoS One. 2019 Feb 11;14(2):e0209905. doi: 10.1371/journal.pone.0209905 (PMC6370198; doi:10.1371/journal.pone.0209905)
Supplement: S1 Table — DNA extracts (4 serial 10x dilutions from each) from cultures of the strains listed demonstrated no signal up to 50 cycles in all cases. Conversely, similar dilutions of extracts from N. meningitidis strains ATCC 53417(A), BAA-335(B), 53414(C), 53419(D), 35559(W), 35560(X) and 35561(Y) consistently amplified after 25 cycles or less. (DOCX) [file pone.0209905.s001.docx]

**S1 Table.** Specificity panel for *sodC* PCR. DNA extracts (4 serial 10x dilutions from each) from cultures of the strains listed demonstrated no signal up to 50 cycles in all cases. Conversely, similar dilutions of extracts from *N. meningitidis* strains ATCC 53417(A), BAA-335(B), 53414(C), 53419(D), 35559(W), 35560(X) and 35561(Y) consistently amplified after 25 cycles or less.

| Species | Strain/source |
| --- | --- |
| *S. agalactiae* | ATCC 12403 (UBI 1931) |
| *S. mitis* | NCTC 10712 (UB 617) |
| *S. epidermidis* | NCTC 11047 (UBI 813) |
| *E. faecalis* | JH2-2 |
| *S. oralis* | NCTC 11427 (UB2 178) |
| *S. aureus* | ATCC 25923 |
| *S. pneumoniae* | ATCC 6303 |
| *S. pyogenes* x 2 | ATCC BAA-1063 and BAA-1064 |
| *S. pseudopneumoniae* | ATCC BAA-960 |
| *S. parasanguinis* | Nasopharyngeal isolate, Coimbra, 2011 |
| *E. coli* | ATCC 25922 |
| *N. lactamica* x 5 | Pharyngeal isolates, Coimbra, 2012 and Bristol, 2015 |
| *N. gonorrhoeae* x 2 | ATCC 31426, oral carriage isolate |
| *P. aeruginosa* | Nasopharyngeal isolate, Coimbra, 2011 |
| *H. parainfluenzae* x 2 | Nasopharyngeal isolates, Coimbra, 2011 and Bristol 2013 |
| *H. influenzae* x 6 | ATCC 10211 and 5 nasopharyngeal isolates, Coimbra, 2011 |
| *M. catarrhalis* | ATCC 25240 |
